# Potentiation of Low-Dose Doxorubicin Cytotoxicity by Affecting P-Glycoprotein through Caryophyllane Sesquiterpenes in HepG2 Cells: An In Vitro and In Silico Study

**DOI:** 10.3390/ijms21020633

**Published:** 2020-01-17

**Authors:** Antonella Di Sotto, Hamid Irannejad, Margherita Eufemi, Romina Mancinelli, Lorena Abete, Caterina Loredana Mammola, Fabio Altieri, Gabriela Mazzanti, Silvia Di Giacomo

**Affiliations:** 1 Department of Physiology and Pharmacology “V. Erspamer”, Sapienza University of Rome, P.le Aldo Moro 5, 00185 Rome, Italy; lorena.abete@uniroma1.it (L.A.); gabriela.mazzanti@uniroma1.it (G.M.); silvia.digiacomo@uniroma1.it (S.D.G.); 2 Department of Medicinal Chemistry, Faculty of Pharmacy, Mazandaran University of Medical Sciences, 48175-866 Sari, Iran; irannejadhamid@gmail.com; 3 Department of Biochemical Science “A. Rossi Fanelli”, Sapienza University of Rome, P.le Aldo Moro 5, 00185 Rome, Italy; margherita.eufemi@uniroma1.it; 4 Department of Anatomical, Histological, Forensic and Orthopedic Sciences, Sapienza University of Rome, P.le Aldo Moro 5, 00185 Rome, Italy; romina.mancinelli@uniroma1.it (R.M.); caterinaloredana.mammola@uniroma1.it (C.L.M.)

**Keywords:** *β*-caryophyllene, *β*-caryophyllene oxide, metronomic treatment, doxorubicin, liver cancer, multidrug resistance, computational chemistry, MDR1 (multidrug resistance-1) inhibition, Structure activity relationship, Active site prediction

## Abstract

Doxorubicin represents a valuable choice for different cancers, although the severe side effects occurring at the high effective dose limits its clinical use. In the present study, potential strategies to potentiate low-dose doxorubicin efficacy, including a metronomic schedule, characterized by a short and repeated exposure to the anticancer drug, and the combination with the natural chemosensitizing sesquiterpenes *β*-caryophyllene and *β*-caryophyllene oxide, were assessed in human hepatoma HepG2 cells. The involvement of P-glycoprotein (P-gp) in the HepG2–chemosensitization to doxorubicin was evaluated. Also, the direct interaction of caryophyllene sesquiterpenes with P-gp was characterized by molecular docking and dynamic simulation studies. A metronomic schedule allowed us to enhance the low-dose doxorubicin cytotoxicity and the combination with caryophyllane sesquiterpenes further potentiated this effect. Also, an increased intracellular accumulation of doxorubicin and rhodamine 123 induced by caryophyllane sesquiterpenes was found, thus suggesting their interference with P-gp function. A lowered expression of P-gp induced by the combinations, with respect to doxorubicin alone, was observed too. Docking studies found that the binding site of caryophyllane sesquiterpene was next to the ATP binding domain of P-gp and that *β*-caryophyllene possessed the stronger binding affinity and higher inhibition potential calculated by MM-PBSA. Present findings strengthen our hypothesis about the potential chemosensitizing power of caryophyllane sesquiterpenes and suggest that combining a chemosensitizer and a metronomic schedule can represent a suitable strategy to overcome drawbacks of doxorubicin chemotherapy while exploiting its powerful activity.

## 1. Introduction

Doxorubicin is an anthracycline antibiotic widely used in the therapy of different cancer types due to its effective cancer-killing potential, mediated by multiple cytotoxic mechanisms involving DNA-damage, blocking of cancer cell growth and increase of intracellular oxidative stress, which in turn lead to destruction of cell structures and cell death [1]. It has also been suggested to be a valuable chemotherapeutic choice for liver cancer, if used through advanced pharmaceutical forms or under polytherapy regimens [2], although sorafenib remains the only approved drug [3]. 

However, the low oral bioavailability of doxorubicin, likely ascribed to the hepatic metabolism and P-glycoprotein (P-gp or MDR1)-mediated intestinal efflux, implies that this drug is mainly administered by a single intravenous injection of high doses [4]. As a consequence, severe dose-dependent side effects on normal tissues, particularly cardiotoxicity and myelosuppression, and multidrug resistance development occur, therefore lowering tolerability and efficacy of doxorubicin chemotherapy [5]. Different strategies have been proposed to overcome these drawbacks. Particularly, suitable formulations or delivery systems to hinder drug degradation and improve its uptake into tumors have been developed. Some of them have been found effective in early stages of advanced hepatocellular carcinoma (HCC), an aggressive malignant tumor, whose effective treatment remains still a great challenge [2]. Although sorafenib is the only approved drug for liver cancer for the early stages of HCC, doxorubicin has been suggested to be a valuable chemotherapeutic choice for liver cancer if used through advanced pharmaceutical forms or under polytherapy regimens [2,6]. 

In order to limit the side effects of the standard chemotherapy protocols and considering the impact of dose and administration scheduling on therapeutic efficacy [7], innovative schedules for cancer treatment have been proposed [8]. Among them, metronomic chemotherapy has been developed as an alternative regimen based on the frequent or continuous administration of low-dose anticancer drugs, without prolonged drug-free periods, whereas the maximum tolerated dose at 2- or 3-week intervals is administered under conventional protocols [9]. This approach enables practitioners to use low drug concentrations while minimizing severe toxicities. Different preclinical studies highlighted a remarkable effectiveness of the metronomic-based chemotherapy in liver cancer, thus suggesting its potential clinical usefulness [10]. 

Increased doxorubicin efficacy has been also reported in combination chemotherapy regimens or multitargeted therapies, based on the use of drug combinations to affect different pathways involved in cancer pathogenesis [11,12]. Analogously, the combination of low-dose cytotoxic drugs and sorafenib or other chemotherapeutics (e.g., paclitaxel, 5-fluorouracil) under metronomic regimens gave interesting results in liver cancer patients [13]. 

Several natural compounds have been also found able to potentiate the effectiveness of conventional anticancer drugs in liver cancer cells and other tumors and to reduce the chemoresistance development [3,14]. Accordingly, we previously highlighted that the natural sesquiterpenes *β*-caryophyllene and its metabolite *β*-caryophyllene oxide produced chemosensitizing effects in different combined anticancer protocols [15,16]. *β*-caryophyllene was also reported to potentiate the antiproliferative activity of paclitaxel [17], whereas *β*-caryophyllene oxide improved the cytotoxic and pro-apoptotic effects of paclitaxel and doxorubicin in human myeloma and human prostate cancer cells [18] and synergized doxorubicin in the inhibition of breast cancer cell proliferation [19]. Furthermore, *β*-caryophyllene oxide was found able to enhance the antiproliferative effects of 5-fluorouracil and oxaliplatin in colon cancer cell lines, partly by the disruption of mitochondrial membrane potential and activation of initiator caspases [20]. 

In line with this evidence, in the present study we assessed possible novel approaches to potentiate the effectiveness of low-dose doxorubicin in liver cancer cells, in order to reduce the drug systemic toxicity. To this end, we scheduled an in vitro metronomic protocol, characterized by a short and repeated exposure to the anticancer drug, as a possible potentiating strategy with respect to a standard long-term exposure. Furthermore, both the long-term and metronomic regimens were applied to the study of the chemosensitizing abilities of the natural sesquiterpenes *β*-caryophyllene and *β*-caryophyllene oxide in combination with doxorubicin. Also, taking into account that P-gp is one of the most recognized hepatic transporters responsible for doxorubicin efflux and its low efficacy [21], the ability of the caryophyllane sesquiterpenes to affect P-gp function and expression were assessed as possible chemosensitizing mechanisms. The in vitro study has been carried out in human hepatoblastoma HepG2 cells, sensitive to doxorubicin and widely used for identifying possible P-gp inhibitors [22,23,24]. In these cells, chemoresistance to doxorubicin has been found mainly mediated by P-gp, with a possible crosstalk between P-gp and MRP-1 [25].

In order to characterize a direct inhibition of P-gp by caryophyllane sesquiterpenes at the binding site and to establish a possible structure activity relationship for the caryophyllane scaffold, an in silico prediction of the interactions between the tested compounds and a previously developed human homology model of P-glycoprotein [26] was also performed. 

## 2. Results

### 2.1. Cytotoxicity of Doxorubicin and Caryophyllane Sesquiterpenes after Long-Term and Metronomic Exposures 

**Figure 1:**
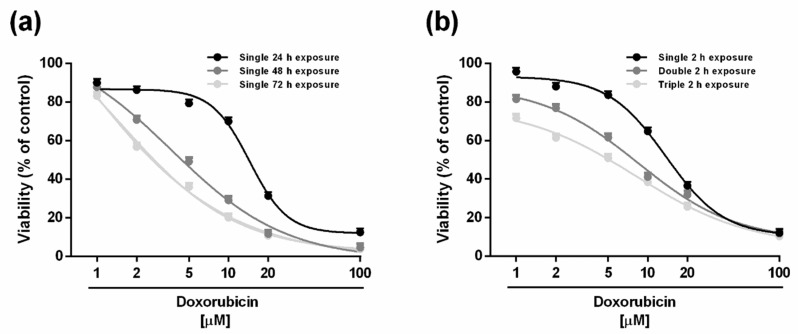
Cytotoxicity of the anticancer drug doxorubicin in HepG2 cells under single long-term exposures of 24, 48 and 72 h (**a**) and under the metronomic schedule. (**b**). In the last protocol, the cells were subjected to a short and/or repeated exposure of 2 h followed by a recovery time of 72 h. Data represent the mean ± SE (standard error) of at least two experiments in which each treatment was tested in triplicate (*n* = 6).

Cytotoxicity of the tested compounds was assessed under different scheduled long-term and metronomic exposure protocols, as reported in the Supplementary Materials (Figures S1 and S2). After a single extended exposure of 24 h, the anticancer drug doxorubicin (concentration range of 1–100 μM corresponding to 0.5–50 μg/mL) exerted early signs of toxicity (about 30% inhibition of cell viability) at 10 µM, achieving the maximum inhibition of 88% at the highest tested concentration of 100 µM (Figure 1a). This effect is significantly increased by time exposure, particularly at low drug concentrations. Indeed, after 48 and 72 h, the anticancer drug produced about a 35% and 60% inhibition of cell viability already at the concentration of 2 µM, which was nontoxic under the 24 h exposure protocol (Figure 1a). According to the IC_50_ values, a long-term exposure of 48 and 72 h increased the doxorubicin potency by about 3-fold and 9-fold, respectively, with respect to the effect obtained after 24 h (Table 1).

Doxorubicin was also assessed under the metronomic protocol, characterized by short (2 h) and/or repeated exposures to the anticancer drug followed by an extended cell recovery time. After a single 2 h exposure, the cytotoxicity profile was similar to that obtained after 24 h, with early toxicity signs (about 35% inhibition of cell viability) at the concentration of 10 μM while achieving the maximum 88% inhibition at 100 μM (Figure 1b). 

Repeated short treatments resulted in a significant increase of the cytotoxicity of low-dose doxorubicin, particularly the triple treatment (Figure 1b). For instance, the lowest-tested concentration of 1 μM was nontoxic in all the experimental conditions except for the tripled short treatment of 2 h which produced about a 30% inhibition of cell viability (Figure 1b). Similarly, the concentration of 5 μM of the anticancer drug resulted in potentiation of about 22% and 33% after a double and triple administration, respectively (Figure 1b). Accordingly, the IC_50_ values of doxorubicin lowered by about 1.2- and 3.1-fold when administered as double and triple short treatments as opposed to a single one (Table 1). The triple short exposure allowed us to achieve an IC_50_ value near to that obtained after a long-term exposure of 48 h and that was significantly lower than that produced after 24 h exposure (Table 1). 

In regard to the natural sesquiterpenes *β*-caryophyllene and *β*-caryophyllene oxide, under the tested concentrations (i.e., 2.5–375 μM corresponding to 0.5–75 μg/mL), they exhibited a similar cytotoxicity profile in all the experimental conditions. Indeed, both compounds produced early toxicity signs (about 20% inhibition of cell viability with respect to vehicle) up to the concentration of 50 μM, whereas biologically significant cytotoxic effects were found starting from the concentration of 125 μM. *β*-caryophyllene was slightly more potent than the epoxide metabolite after the long-term 48 and 72 h exposures, with IC_50_ values about 1.3-fold lower than those of *β*-caryophyllene oxide; conversely, similar values were obtained after 24 h exposure (Table 1). Under the metronomic exposures the compounds exhibited similar cytotoxicity profiles, with a slightly increased potency of about 1.4-fold after repeated treatments in respect to a single short exposure of 2 h (Table 1). However, the cytotoxicity power of both compounds under metronomic treatments was lower than that found after long-term exposures. 

### 2.2. Chemosensitizing Effects of Caryophyllane Sesquiterpenes in Combination with Doxorubicin under Long-Term and Metronomic Exposures 

**Figure 2:**
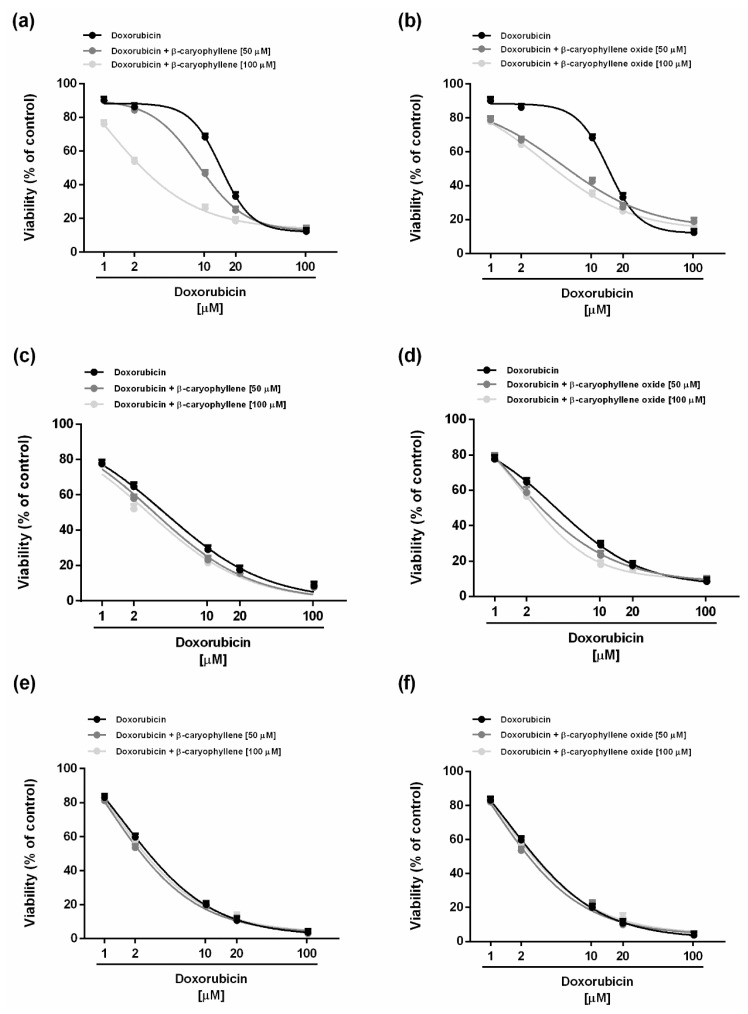
Cytotoxicity of doxorubicin in combination with the sesquiterpenes *β*-caryophyllene (**a**,**c**,**e**) and *β*-caryophyllene oxide (**b**,**d**,**f**) after standard long-term treatments in HepG2 cells. (**a**,**b**) Single exposure of 24 h. (**c**,**d**) Single exposure of 48 h. (**e**,**f**) Single exposure of 72 h. Data represent the mean ± SE (standard error) of at least two experiments in which each treatment was tested in triplicate (*n* = 6).

The chemosensitizing properties of caryophyllane sesquiterpenes in combination with the anticancer drug doxorubicin were assessed under both long-term and metronomic schedules. According to previous published criteria for studying the chemosensitizing properties of a chemical [27], the low cytotoxic concentrations (less than 20% inhibition of cell viability) of 50 and 100 μM (corresponding to 10 and 20 µg/mL) of the natural sesquiterpenes *β*-caryophyllene and *β*-caryophyllene oxide were selected for the combination experiments. Under our experimental conditions, both the sesquiterpenes were able to potentiate the effect of doxorubicin in different experimental conditions, although with different profiles. Particularly, after 24 h exposure, the lower chemosensitizing concentration (50 μM) of *β*-caryophyllene significantly enhanced the doxorubicin-cytotoxicity starting from the concentration of 10 µM, achieving almost the maximum potentiation of about 32% at concentration of 20 µM (Figure 2a). 

Under the same experimental conditions, *β*-caryophyllene oxide produced a significant increase (about 20%) of doxorubicin cytotoxicity at the lowest concentration of 1 µM, reaching the highest potentiation of 40% at the concentration of 10 µM (Figure 2b). Surprisingly, at the higher concentration of 100 µM, *β*-caryophyllene was more effective than *β*-caryophyllene oxide as a chemosensitizing agent, producing a potentiation of about 42% already at concentration of 2 µM, in spite of a 25% increase obtained with the epoxide metabolite (Figure 2a,b). 

The IC_50_ value of doxorubicin was lowered by about 14.4-fold and 4.2-fold in combination with the higher chemosensitizing concentration (100 µM) of *β*-caryophyllene and *β*-caryophyllene oxide, respectively (Table 2). On the basis of these results, *β*-caryophyllene seems to be more effective as a chemosensitizer than the epoxide metabolite at higher chemosensitizing concentrations, in spite of a lower power at the concentration of 50 µM. 

After 48 and 72 h exposures, the chemosensitizing power of both sesquiterpenes towards doxorubicin disappeared, with the cytotoxicity of the combination being quite similar to that of the anticancer drug alone (Figure 2c–e). Accordingly, the IC_50_ value of doxorubicin was slightly affected by the combinations (Table 2). 

**Figure 3:**
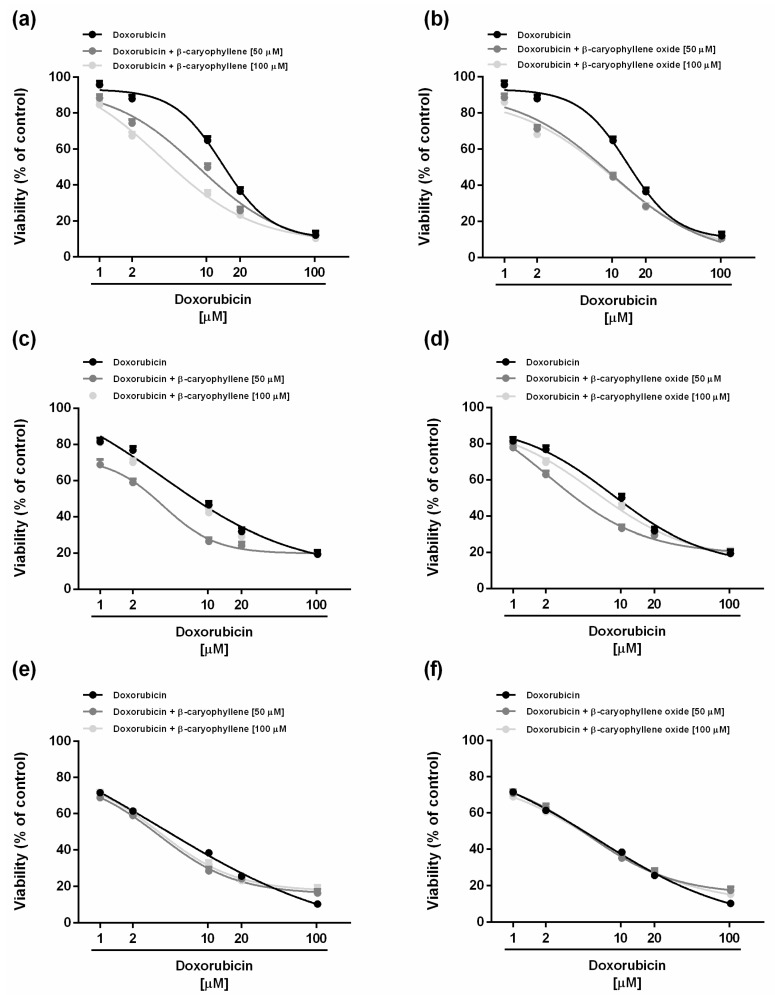
Cytotoxicity of doxorubicin in combination with the sesquiterpenes *β*-caryophyllene (**a**,**c**,**e**) and *β*-caryophyllene oxide (**b**,**d**,**f**) after metronomic exposures in HepG2 cells. (**a**,**b**) Single short exposure of 2 h. (**c**,**d**) Double repeated exposure of 2 h followed by a recovery time of 72 h. (**e**,**f**) Triple repeated exposure of 2 h followed by a recovery time of 72 h. Data represent the mean ± SE (standard error) of at least two experiments in which each treatment was tested in triplicate (*n* = 6).

When assessed under metronomic conditions, both sesquiterpenes were able to enhance the cytotoxicity of doxorubicin in a similar manner after a single short exposure of 2 h (Figure 3). 

For instance, when combined with the lower chemosensitizing concentration (50 µM) of the caryophyllane sesquiterpenes, the doxorubicin concentration of 2 µM produced a 35% cytotoxicity, in spite of a null effect of the only anticancer drug (Figure 3a,b). A similar behavior was observed at progressively increasing doxorubicin concentrations, at which a potentiation from 10% to 18% occurred (Figure 3a,b).

Moreover, combining doxorubicin with the higher chemosensitizing concentration (100 μM) of *β*-caryophyllene further enhanced its cytotoxicity with respect to the lower chemosensitizing dose, in spite of a null potentiation produced by *β*-caryophyllene oxide (Figure 3a,b). Particularly, *β*-caryophyllene 100 µM produced a potentiation from 10% to 37% of low concentrations (i.e., 1–10 μM) of doxorubicin. The IC_50_ value of doxorubicin reduced by 1.6-fold to almost 4-fold in combination with *β*-caryophyllene, whereas it reduced by about 2-fold in combination with *β*-caryophyllene oxide (Table 3).

After the double repeated exposure, the highest potentiation (almost 4-fold) of doxorubicin cytotoxicity was obtained in combination with *β*-caryophyllene oxide at the chemosensitizing concentration of 50 µM, followed by the same concentration of *β*-caryophyllene (almost 3-fold potentiation in respect to doxorubicin); conversely, the chemosensitizing concentration of 100 µM of both sesquiterpenes enhanced the doxorubicin cytotoxicity by at least 1.5-fold (Figure 3c,d; Table 3). The triple repeated exposure only slightly increased the cytotoxicity of doxorubicin in combination with *β*-caryophyllene (about 1.5-fold potentiation with respect to doxorubicin), without effects of *β*-caryophyllene oxide (Figure 3e,f; Table 3).

On the basis of the obtained results, both sesquiterpenes displayed chemosensitizing effects, particularly after the single treatment of 24 h and a single or double short-exposure protocol. Therefore, the above-mentioned time exposures were chosen to characterize the interaction nature between doxorubicin and caryophyllane sesquiterpenes. 

According to Di Giacomo et al. [27], a combination index (CI) value lower than 1 highlights a synergistic interaction, while an additive effect occurs when this value is equal to 1; conversely, if CI is higher than 1, the interaction is considered antagonistic. 

Under our experimental conditions, the long-term exposure of 24 h of the combined treatments produced CI values of 0.85 and 0.58, and of 0.61 and 0.75 for doxorubicin plus *β*-caryophyllene or *β*-caryophyllene oxide 50 and 100 µM, respectively. Similar results were obtained after a single short exposure of 2 h, being the CI values of 0.73 and 0.52 and of 0.62 and 0.91 for doxorubicin plus *β*-caryophyllene or *β*-caryophyllene oxide 50 and 100 µM, respectively. After the double short-exposure protocol, the CI values were 0.58 and 0.94, and 0.44 and 1.02 for doxorubicin plus *β*-caryophyllene or *β*-caryophyllene oxide 50 and 100 µM, respectively. At last, after the triple short-exposure protocol, the CI values were 0.75 and 0.99 for doxorubicin plus *β*-caryophyllene 50 and 100 µM, but 0.99 and 1.3 for doxorubicin plus *β*-caryophyllene oxide 50 and 100 µM.

**Figure 4:**
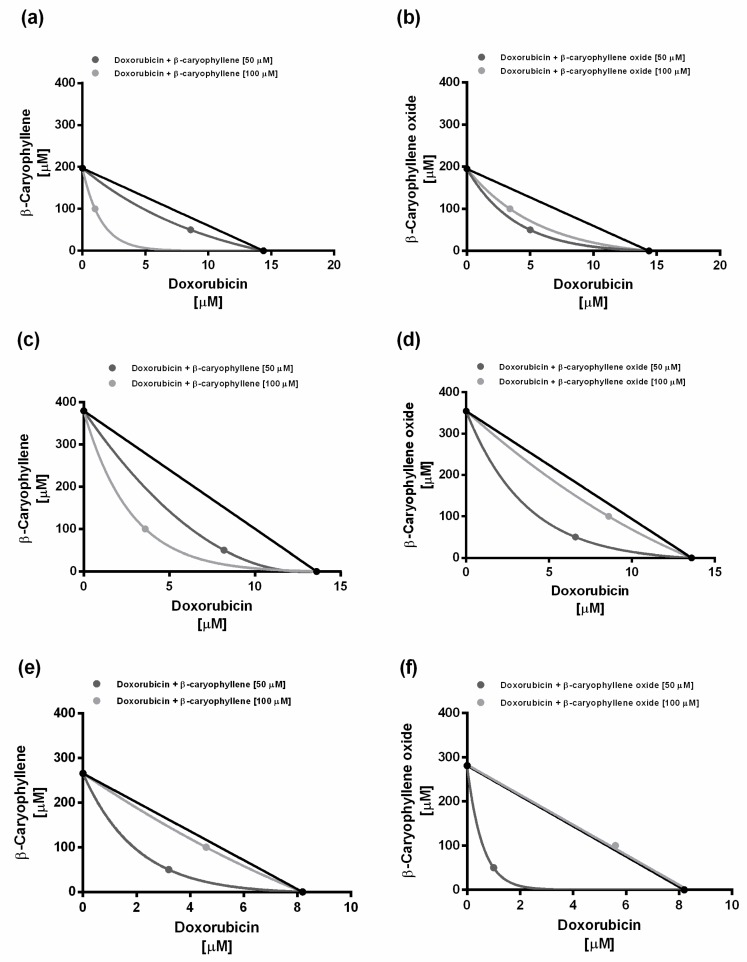
Isobolographic analysis of the cytotoxic effect obtained with the combination of doxorubicin and *β*-caryophyllene or *β*-caryophyllene oxide at the chemosensitizing concentrations of 50 and 100 µM in HepG2 cells. (**a**,**b**) Long-term exposure of 24 h. (**c**,**d**) Short exposure of 2 h. (**e**,**f**) Double exposure of 2 h.

On the basis of these results, the interaction between doxorubicin and caryophyllane sesquiterpenes appears to be mainly due to synergistic mechanisms. The isobologram analysis was in agreement with the CI values and highlighted prevailing synergistic effects of the caryophyllane sesquiterpenes with doxorubicin, except for the high chemosensitizing concentration of *β*-caryophyllene oxide after two repeated exposures of 2 h, which seems to produce an additive effect (Figure 4).

### 2.3. Caryophyllane Sesquiterpenes Affect Doxorubicin and Rhodamine Accumulation in HepG2 Cells

**Figure 5:**
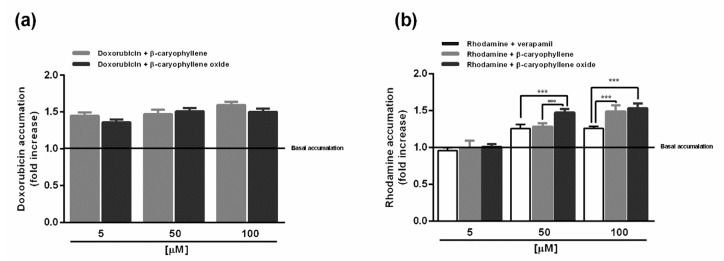
Effect of the sesquiterpenes *β*-caryophyllene and *β*-caryophyllene oxide on intracellular accumulation of doxorubicin (**a**) and rhodamine 123 (**b**) in HepG2 cells. The basal accumulation indicates that found in the vehicle control. *** *p* < 0.001 (t-Student test), significantly higher than verapamil. °°° *p* < 0.001 (*t*-Student test), significantly higher than *β*-caryophyllene. Data represent the mean ± SE (standard error) of at least two experiments in which each treatment was tested in triplicate (*n* = 6).

In order to determine whether caryophyllane sesquiterpenes potentiated the cytotoxic effects of doxorubicin by enhancing its intracellular accumulation, specific flow cytometric analyses were carried out. To this end, the doxorubicin concentration of 20 µM, which induced submaximal cytotoxic effects in all the experimental conditions, was selected to be tested in the presence of the caryophyllane sesquiterpenes (concentrations 5, 50 and 100 µM) under the short-term exposure of 2 h, during which a synergistic effect was already observed. In comparison with vehicle, both the sesquiterpenes similarly increased the doxorubicin accumulation at all the tested concentrations, achieving a maximum increase of 60% and 50% with *β*-caryophyllene and *β*-caryophyllene oxide, respectively (Figure 5a). 

Subsequently, considering that the P-gp pump is the most recognized hepatic transporter responsible for doxorubicin efflux and lowered efficacy [21], the substances were also assessed in the same experimental conditions for the accumulation of rhodamine 123, used as a more specific substrate for P-glycoprotein [28]. Our results highlighted that, despite a null effect of the lowest concentration of 5 µM, both the sesquiterpenes and verapamil increased rhodamine accumulation at the higher concentrations of 50 and 100 µM (Figure 5b). Particularly, the increased rhodamine accumulation induced by sesquiterpenes appeared to be concentration-dependent, with a higher efficacy of *β*-caryophyllene oxide (47% increase vs. control) compared to *β*-caryophyllene (29% increase vs. control) at the low chemosensitizing concentration of 50 µM, despite a similar effect at 100 µM (about 50% increase vs. control). Interestingly, the sesquiterpenes exerted a similar or higher activity than the standard verapamil.

On the basis of the accumulation assay results, the combined treatment of doxorubicin and caryophyllane sesquiterpenes seems to affect the efflux pumps in HepG2 cells. Accordingly, our previous study highlighted that caryophyllane sesquiterpenes affected the efflux of sorafenib, mainly mediated by MRP1 and MRP2 and partly by MDR1, in Alexander and Hepa 1–6 cells [16].

Also, the increased accumulation of rhodamine 123 in the presence of tested sesquiterpenes enable us to hypothesize that, under our experimental conditions, *β*-caryophyllene and *β*-caryophyllene oxide can mainly act by interfering with the P-gp transporter. This hypothesis is also supported by our previous data about the lack of chemosensitizing effects of caryophyllane sesquiterpenes when assessed in combination with cisplatin (Figure S3), whose cellular efflux seems to be mainly mediated by copper-transporting P-type adenosine triphosphatases ATP7A and ATP7B [29] and favored by increased intracellular pH [30]. 

### 2.4. Caryophyllane Sesquiterpenes Affect the Doxorubicin-Mediated Increase of P-gp Expression in HepG2 Cells

In line with the above reported results, the expression of the P-gp protein was detected by western blotting and immunofluorescence. According to the above reported protocol, the HepG2 cells were treated for 2 h with doxorubicin (20 μM) both alone and in combination with caryophyllane sesquiterpenes at the lowest effective chemosensitizing concentration of 50 μM. 

**Figure 6:**
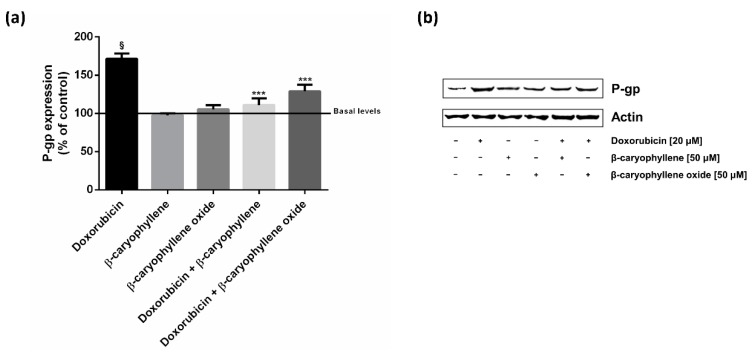
Effect of the sesquiterpenes *β*-caryophyllene and *β*-caryophyllene oxide on P-gp expression induced by doxorubicin in HepG2 cells. The cells were exposed to caryophyllane sesquiterpenes (50 µM), doxorubicin (20 µM) or their combination for 2 h. Then, the pellets were harvested for the western blotting analysis. The basal level represents the P-gp expression in the vehicle control. (**a**) Densitometric bar graph analysis obtained from at least two independent replicates. Data are expressed as mean ± standard error. (**b**) Representative western blotting image showing the expression levels of P-gp and *β*-actin used as protein loading control. *** *p* < 0.001 (*t*-Student test), significantly lower than doxorubicin. ^§^ *p* < 0.001 (*t*-Student test), significantly higher than the vehicle control (basal level).

Western blotting analysis revealed a marked increase of P-gp expression (about 70% increase compared with control) due to doxorubicin treatment, which was significantly reduced by about 60% and 40% in combination with *β*-caryophyllene and *β*-caryophyllene oxide, respectively (Figure 6). When the cells were treated with only sesquiterpenes, the P-gp expression was strictly similar to that of the vehicle control (Figure 6).

**Figure 7:**
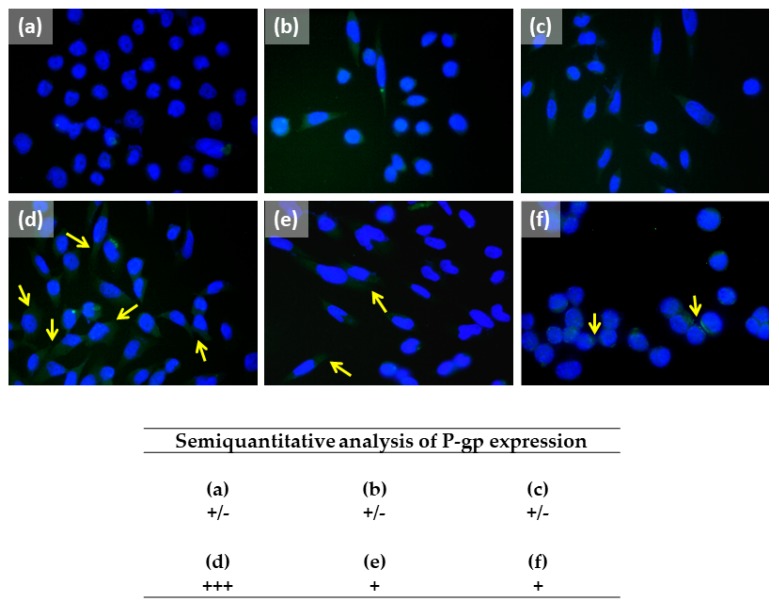
Representative immunofluorescence (IF) images and semiquantitative analysis of P-glycoprotein (P-gp) expression in HepG2 cells (OM 40×). The cells were exposed to a short treatment of 2 h with caryophyllane sesquiterpenes (50 µM), doxorubicin (20 µM) or their combination, then fixed by methanol. Yellow arrows indicate the presence of P-gp protein. (**a**) Vehicle control (EtOH 1 % *v*/*v*). (**b**) *β*-Caryophyllene. (**c**) *β*-Caryophyllene oxide. (**d**) Doxorubicin. (**e**) Combination of doxorubicin and *β*-caryophyllene. (**f**) Combination of doxorubicin and *β*-caryophyllene oxide. The semiquantitative analysis has been carried out (four fields for each treatment) using a previous published grading system [31]: 0%–5% = negative; 6%–10% = +/−; 11%–30% = +; 31%–60% = ++; >61% = +++.

At the immunofluorescence analysis, the cells treated with doxorubicin displayed a significant increased expression in P-gp compared the control cells (Figure 7a,d). By contrast, the presence of P-gp was downregulated in the combinations of the anticancer drug and both the caryophyllane sesquiterpenes with respect to doxorubicin (Figure 7d–f). The sesquiterpenes alone did not affect P-gp expression (Figure 7a–c).

### 2.5. In Silico Prediction of the Possible Interaction between Caryophyllene Sesquiterpenes and P-Glycoprotein

The possible direct interaction between caryophyllane sesquiterpenes and P-gp proteins was screened by molecular docking, using a previous published human homology model of P-glycoprotein [26]. The sesquiterpene *α*-caryophyllene (also known as *α*-humulene), a ring-opened isomer of *β*-caryophyllene, was also included in the in silico analysis in order to characterize possible specific features of the caryophyllane scaffold for the interaction with P-gp structure. Verapamil was included as positive control to validate the computational protocol and to predict the potency of tested sesquiterpenes as P-gp inhibitors. 

**Figure 8:**
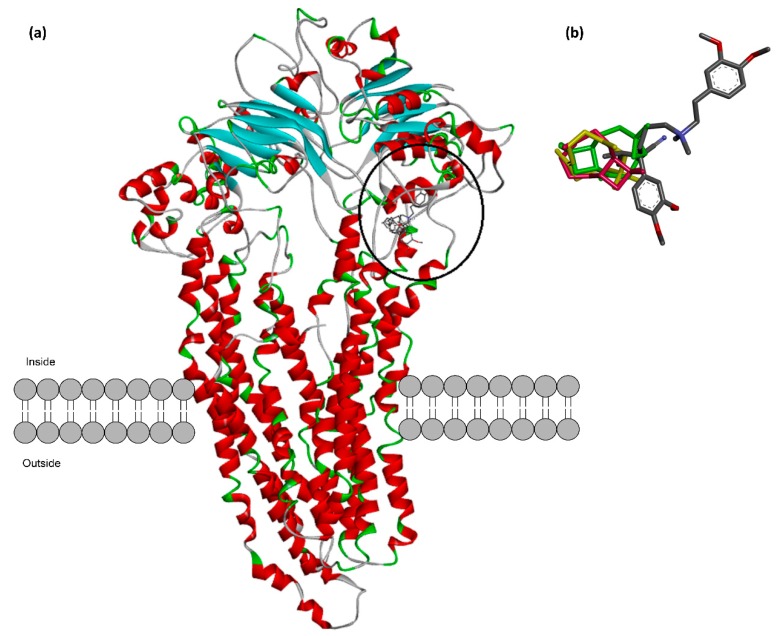
Predicted active site has been circled in the figure for the caryophyllane sesquiterpenes. (**a**) The common binding site predicted for verapamil and the caryophyllane sesquiterpenes is shown as circled. (**b**) Binding conformation of verapamil (colored by atom), *α*-caryophyllene (yellow), *β*-caryophyllene (red) and *β*-caryophyllene oxide (green).

The predicted binding sites for the caryophyllane sesquiterpenes were based on the calculated binding energies in the output docking clusters. Accordingly, the best docking score for *α*-caryophyllene, *β*-caryophyllene and *β*-caryophyllene oxide was found in a hydrophobic space next to the nucleotide binding domain mostly covered by Leu225, Phe760, Leu781, Phe771, Ile 228, Met763 and Val231 (Figure 8a). A particular image of the binding conformation for the caryophyllene sesquiterpenes and verapamil in the predicted binding site is illustrated in Figure 8b. The tested sesquiterpenes were found to bind in the same location next to the ATP binding domain. Surprisingly, this binding location is the same as the binding site predicted for verapamil in a recently reported study [26]. 

The calculated binding energy and its decomposition values for the caryophyllane sesquiterpenes and the standard P-gp inhibitor verapamil were calculated by MM-PBSA method and are displayed in Table 3. Among sesquiterpenes, *β*-caryophyllene was found to possess the lowest binding energy (−88.46 kJ/moL), followed by *β*-caryophyllene oxide (−82.57 kJ/moL) and *α*-caryophyllene (−73.01 kJ/moL). This means that it is characterized by a stronger binding affinity and a higher inhibition potential of *β*-caryophyllene towards the predicted binding site of P-gp. As expected, the low binding energy (−142.97 kJ/moL) of verapamil confirms its high inhibition power (Table 3). 

**Figure 9:**
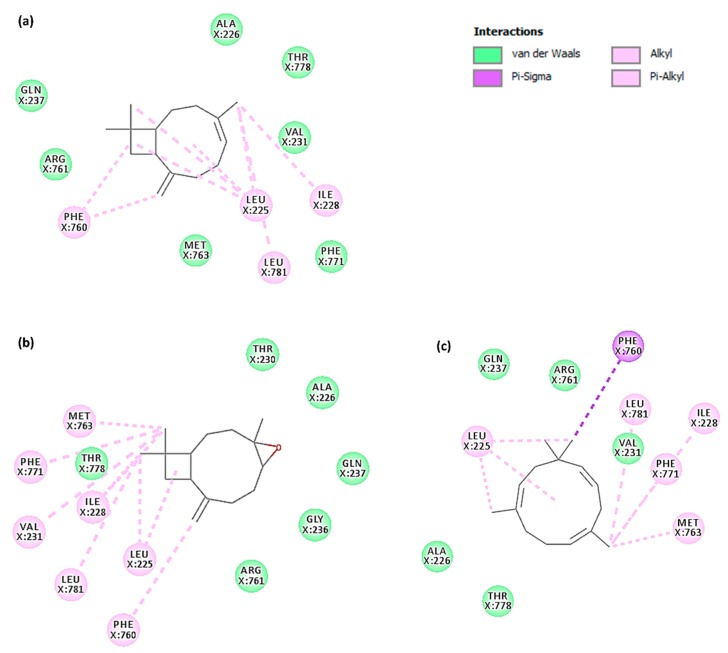
2D-map of *β*-caryophyllene (**a**) and *β*-caryophyllene oxide (**b**), in comparison with the ring-opened isomer *α*-caryophyllene (**c**), in the binding site of P-gp representing interactive amino acids and types of interactions.

Investigation of the binding interactions (Figure 9) showed that the nonpolar dimethylcyclobutane moiety of the structure is capable of forming several favorable hydrophobic interactions. This moiety is not present in the structure of *α*-caryophyllene and probably this extra-motif accounts for the higher binding affinity of *β*-caryophyllene and *β*-caryophyllene oxide compared to α-caryophyllene. A high positive value of electrostatic energy for *α*-caryophyllene (23.59 kJ/moL), as denoted in Table 3, is the result of lack of the latter hydrophobic structural motif in the structure of *α*-caryophyllene. Thereupon, polar solvation energy of *α*-caryophyllene is higher than the other caryophyllane sesquiterpenes. 

**Figure 10:**
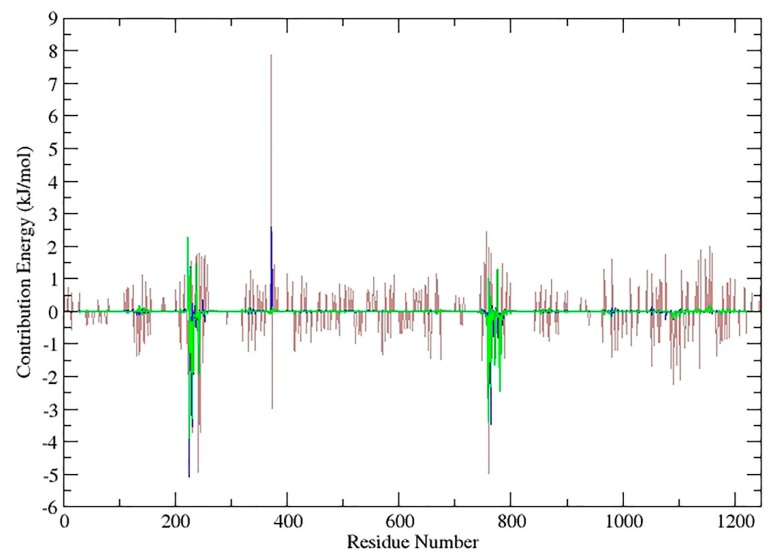
An overlay graph of contribution energy per residue to the binding energy (kJ/moL) for the three caryophyllane sesquiterpenes. Brown line represents contribution energy of *α*-caryophyllene, blue line represents *β*-caryophyllene and green line denotes *β*-caryophyllene oxide.

The 2D-map of caryophyllane sesquiterpenes (Figure 9), representing the binding conformation of the tested compounds in the predicted binding site of P-gp, also displays the amino acid residues involved in the interaction and shows that nonpolar interactions such as van der Waals and pi-alkyl are exclusively formed. The contribution energy per residue to the binding energy showed that α-caryophyllene has the worst interaction pattern since there are a lot of positive values (Figure 10; brown line) in the energy of interacting amino acids with the ligand. Moreover, *β*-caryophyllene (Figure 10; blue line) has more negative and less positive values in interacting amino acids than *β*-caryophyllene oxide (Figure 10; green line).

The exact values for contribution energy of each amino acid to the binding energy of *β*-caryophyllene, *β*-caryophyllene oxide and verapamil are reported in Table S1, whilst those of α-caryophyllene were not included due to the much more unfavored positive values. Particularly, polar residues, such as Glu223, Gln237, Lys372 and Arg761, are found as the main unfavored interacting amino acids, giving positive energy values to *β*-caryophyllene and *β*-caryophyllene oxide binding. 

Actually, nonpolar and hydrophobic residues have the main role in increasing binding affinity of the caryophyllane sesquiterpenes. The most important amino acids were found to be Leu225, Ile228, Val231, Phe760, Leu764, Phe771, and Leu781, which are naturally hydrophobic (Figure 9; Table S1). In regard to verapamil, Glu223 was similarly found to have a highly positive interaction energy of +16.3 kJ/mol and the main favorable interacting amino acids were Val231, Leu241, Tyr244, Phe760, Leu764, and Phe1090 (Figures S4 and S5; Table S1).

## 3. Discussion

Caryophyllane sesquiterpenes are natural phytochemicals widely occurring in terrestrial plants and plant-associated fungal species [32,33,34,35,36,37], and are present in marine environments as secondary metabolites from the resident living organisms and microbes [38,39,40,41]. 

From a chemical point of view, *β*-caryophyllene and *β*-caryophyllene oxide are characterized by the caryophyllane skeleton, a unique bicyclic structure with a rare dimethylcyclobutane ring fused in a trans configuration to a nine-carbon ring containing a 1,5-diene [42]. 

These phytochemicals have attracted greater attention by researchers due to their multitarget and pleiotropic bioactivities along with a safety profile [43,44,45]. Accordingly, *β*-caryophyllene is known to bind to the CB2 receptor as an agonist and to induce anti-inflammatory, analgesic and neuroprotective effects [46,47,48]. Furthermore, both sesquiterpenes have been highlighted to possess interesting chemopreventing properties, due to their ability to counteract cell injury induced by environmental pollutants [49,50,51,52] and to inhibit cancer cell growth and proliferation, through affecting several key pathways for cancer development, such as mitogen-activated protein kinase (MAPK), PI3K/AKT/mTOR/S6K1 and STAT3 pathways [47,53,54,55,56]. Also, they were reported to potentiate the efficacy of some anticancer drugs, acting as chemosensitizers [15,16,17,19,20].

In our previous study, *β*-caryophyllene oxide was found to produce chemosensitizing effects towards sorafenib in hepatocellular carcinoma cells by inhibiting the efflux through MDR1 (P-gp), MRP1 and MRP2 pumps [16]. 

In line with this evidence and in trying to find alternative effective and low-toxic therapeutic strategies for liver cancer management, in the present study we evaluated the chemosensitizing power of caryophyllane sesquiterpenes in combination with doxorubicin in human hepatoma cells by applying different administration schedules, based on both single long-term and short and repeated exposures. These protocols were scheduled to be representative of standard chemotherapy, based on the administration of the maximum tolerated doses and associated with several side effects and chemoresistance development, and of the metronomic regimen suggested to be a safe and potentially useful alternative strategy for different cancers, among which is advanced unresectable hepatocellular carcinoma [10].

According to the literature [22,57], our results showed that the cytotoxicity of doxorubicin increased with the exposure time, as shown by the IC_50_ reduction, by 4-fold and 9-fold after the treatments of 48 h and 72 h, respectively, with respect to 24 h. Moreover, metronomic conditions allowed us to achieve a cytotoxicity similar to that found after 48 h, mainly increasing the efficacy of low-dose doxorubicin. 

Previous studies also showed that severe adverse effects and efficacy of doxorubicin chemotherapy can be relieved by choosing an optimal dosing schedule, based on dosage, interval and time exposure in mice [58]. Moreover, the metronomic administration of a non-pegylated liposomal doxorubicin formulation in advanced breast cancer patients was a feasible and attractive alternative to the classic protocol, being clinically effective and safe [59,60,61]. 

Accordingly, Riganti et al. [62] highlighted an increased efficacy of two-repeated low-dose doxorubicin in vitro and in vivo against P-gp-expressing drug-resistant tumors, with respect to a standard treatment with a single high dose, without greater side effects. Interestingly, this effect was linked to a higher intracellular oxidative stress, ascribed to an impairment of the mitochondrial respiratory chain and reduced ATP synthesis, which in turn leads to increased lipid peroxidation, reduction of GSH levels and activation of necro-apoptotic mechanisms. 

Under our experimental conditions, we also found that doxorubicin cytotoxicity can be further potentiated in combination with caryophyllane sesquiterpenes, which mainly synergized its low doses. The chemosensitizing properties of *β*-caryophyllene and *β*-caryophyllene oxide towards doxorubicin were highlighted under both a single 24 h long-term exposure and metronomic conditions, whereas no effects were displayed in the other long-term protocols. 

This behavior suggests that long-term exposure can negatively affect the chemosensitizing properties of caryophyllane sesquiterpenes. Particularly, their metabolic biotransformation into ineffective metabolites can be expected. 

The metabolic fate of *β*-caryophyllene and *β*-caryophyllene oxide has been poorly investigated. Asakawa et al. [63,64] found that, when the compounds were orally administered in rabbits, (10S)-(-)-14-hydroxycaryophyllene-5,6-oxide was the main metabolite of both *β*-caryophyllene and *β*-caryophyllene oxide; (-)-caryophyllene-5,6-oxide-2,12-diol was isolated as an additional minor metabolite of β-caryophyllene. The metabolic fate of these sesquiterpenes in human cells and the impact on the bioactivity loss requires characterization. 

On the basis of these results, the two repeated short treatments, along with the long-term exposure of 24 h, appear the most suitable experimental conditions to exploit the chemosensitizing properties of the caryophyllane sesquiterpenes towards doxorubicin in HepG2 cells. Further studies will allow researchers to confirm the in vivo effectiveness of these combinations and to evaluate a possible interest for further pharmacological applications.

Riganti et al. [62] also highlighted that administering two-repeated low-dose doxorubicin was more effective in drug-resistant P-gp-positive cancer cells than drug-sensitive P-gp-negative cancer cells. However, this schedule did not affect the expression of the P-gp transporter, known to be involved in cellular doxorubicin efflux and drug resistance. In line with this evidence and taking into account our previous study displaying caryophyllane sesquiterpenes to affect the P-gp-mediated efflux in cancer cells [15,16], we also assessed the effect of the combined treatments on this transporter in hepatoma HepG2 cells and the possible involved mechanisms.

P-gp, also known as MDR1 or ABCB1, is an energy-dependent drug efflux pump, encoded by the human *mdr1* gene, and plays a pivotal role in drug pharmacokinetics and permeability [65]. Its overexpression makes some tumors resistant to anticancer drugs, due to their decreased intracellular accumulation, thus suggesting it could represent a possible strategy to reverse cancer multidrug resistance [66]. Structurally, it is a 170 kDa surface glycoprotein, with two bundles of six transmembrane domains, separated by intracellular loops, containing the ATP-binding sites, namely nucleotide-binding domains (NBD). 

Each P-gp protein contains two ATP-binding domains, namely NBD1 and NBD2, which represent its power units since they transfer energy to transport the substrates across the membranes. Each NBD comprises three segments: A Walker A motif for the ATP-binding, a Walker B domain for magnesium ions, which contribute to stabilization of the ATP-binding site, and a signature C motif, which accelerates ATP hydrolysis through chemical transition. It also seems involved in the transduction of the energy from ATP hydrolysis to conformational changes in the transmembrane domains, required for the substrate translocation [67]. 

Typically, P-gp transports lipophilic compounds, which accumulate within the lipid bilayer and amphipathic molecules, aligned in the interfacial region [68]. The process requires drug identification by P-gp, followed by ATP binding and hydrolysis; the released energy, in turn, can be used to transport the chemical against the concentration gradient, through the central pore of the pump, outside the cell [69]. 

The true mechanism responsible for drug efflux through P-gp has been explained in different models, according to which drug can directly interact with the transporter, thus being pumped outside of the cell (classical pump model) or with the lipid biomembrane and then can be moved directly into the extracellular space or flipped by the transporter from the inner leaflet towards the outer leaflet of biomembrane (flippase model). Another model proposed P-gp to be a “vacuum cleaner”, responsible for the removal of lipophilic compounds partitioned into the membrane [68]. 

The inhibition of P-gp function can occur through different mechanisms, including a competitive, noncompetitive or allosteric inhibition for the binding site, an interference with ATP hydrolysis and a change in the phospholipid membrane integrity and fluidity [70]. 

Some inhibitors have been found to affect P-gp expression, thus counteracting its upregulation due to drug inducers or cancer resistance [68]. Also, affecting intracellular calcium homeostasis has been highlighted to be connected to P-gp expression or function, although the true mechanisms are still unknown [71]. For instance, verapamil is known to competitively inhibit P-gp by blocking the binding sites and to alter its expression, whereas quercetin has been reported to block ATP hydrolysis and surfactants that affect integrity of membrane lipids, thus inducing modifications of the P-gp secondary and tertiary structure and ultimately the loss of function [72]. 

Eid et al. [73] hypothesized that lipophilic terpenoids, such as thymol, menthol, aromadendrene, *β*-sitosterol-O-glucoside and *β*-carotene, can act as competitive inhibitors of P-gp in tumor cells.

Under our experimental conditions, *β*-caryophyllene and *β*-caryophyllene oxide were found to act as possible P-gp- inhibitors in HepG2 cells, since they increased doxorubicin accumulation and more specifically interfered with the P-gp-mediated rhodamine efflux. This behavior agrees with previous evidence in leukemic and hepatocarcinoma cells [15,16]. 

Moreover, results of the docking study showed that a hydrophobic space next to the nucleotide-binding domain of P-gp was bound by the caryophyllane sesquiterpenes, mainly through nonpolar and hydrophobic residues. *β*-caryophyllene and *β*-caryophyllene oxide appear to bind more tightly to P-gp with respect to α-caryophyllene, thus suggesting that the *trans*-configuration of the caryophyllane scaffold is a key feature for achieving a tight binding with the transporter. Also, the nonpolar dimethylcyclobutane moiety of the structure is capable of forming several favorable hydrophobic interactions. To the best of our knowledge, this is the first study highlighting a structure-activity relationship for the caryophyllane sesquiterpenes as ABC transporter inhibitors. 

The standard P-gp inhibitor verapamil binds to the same site of caryophyllane sesquiterpenes although with higher strength, as displayed by binding energies, thus suggesting a greater inhibitory power. However, results of the accumulation assay highlighted that the tested compounds inhibited rhodamine 123 efflux with a similar or slightly greater potency than verapamil, leading to the hypothesis that multiple mechanisms could be responsible for P-gp inhibition by caryophyllane sesquiterpenes.

Legault et al. [17] hypothesized that *β*-caryophyllene could be accumulated in the tumor cell membrane, altering its permeability and increasing the intracellular accumulation of anticancer drugs, with consequent strengthening of their activity. Accordingly, Di Giacomo et al. [15,16] suggested that caryophyllane sesquiterpenes were able to interfere with ABC-mediated transport, likely due to their lipophilic nature. Indeed, lipophilic compounds can interact directly with P-gp by forming hydrogen and ionic bonds with side chains of amino acids of the protein, thus interfering with the 3D structure of P-gp (conformation) and inhibiting activity [74]. 

Indeed, *β*-caryophyllene is known to be a lipophilic molecule with very low water solubility and to possess a great capacity to interact with membrane phospholipids and to be partitioned in the lipid bilayer, altering the cooperativity between phospholipids and between phospholipids and transporter proteins, thus likely affecting their function [75]. Recently, this hypothesis has been confirmed in a membrane model of soybean phosphatidylcholine, in which *β*-caryophyllene, at high concentrations, was found to be retained and embedded in the liposome bilayer, likely due to a stable interaction with the phospholipid: this effect was similar to that induced by cholesterol, known to alter membrane packing, order, and fluidity [55]. 

Several biochemical and biophysical studies suggested a complex interplay between P-gp and the lipid environment, which can be reflected in the modulation of protein function. Membrane lipid composition and fluidity, phospholipid headgroup, and acyl chain length are revealed to be key factors in the control of drug-binding and transport [68]. Moreover, the presence of cholesterol-rich microdomains in the biomembrane has been reported to affect P-gp-function, although the true mechanisms remain to be clarified [68]. For instance, including 30% cholesterol in DMPC proteoliposomes reduced the affinity binding of P-gp for ATP [76]. The presence of cholesterol has been also reported to reduce drug-binding affinity, particularly of large substrates [77]. 

Under our experimental conditions, the caryophyllane sesquiterpenes also affected the increased expression of P-gp induced by doxorubicin, as highlighted by both western blotting and immunofluorescence analysis. P-glycoprotein expression may be dynamically controlled by physiological stimuli and the upregulation seems to occur only in cells where there is basal level of expression. Also, there are several stress response pathways, among which are Raf kinase, protein kinase C, epidermal growth factor (EGF), fos and NF-kB [78]. For instance, it is thought that EGF stimulation can lead to phospholipase C activity which in turn activates PKC, upregulating the function of P-glycoprotein via phosphorylation [79]. 

The *mdr1* gene has also been found to be transcriptionally regulated by the signal transducer and activator of transcription 3 (STAT3), which binds with its promoter sequence [80]. In support, STAT3 inhibition has been found to be associated with a mdr1 mRNA down-regulation in ovarian cancer cells [81]. Accordingly, *β*-caryophyllene was previously reported to affect the STAT3 phosphorilation at tyrosine 705 residue. Also, *β*-caryophyllene oxide suppressed constitutive STAT3 activation in multiple myeloma cancer cells [82]. This evidence suggests that inhibiting the activation of STAT3 pathways represents a possible mechanism accounting for the inhibition of P-gp expression by caryophyllane sesquiterpenes. 

This evidence supports the hypothesis that caryophyllane sesquiterpenes can affect P-gp by multiple inhibitory mechanisms, including a direct efflux inhibition, by blocking transporters at the binding site, an indirect interference with its active protein conformation, due to an alteration of the membrane permeability, and a modulation of the protein expression, likely affecting its transcriptional regulation. Further studies will allow us to better characterize the mechanisms of ABC transporter inhibition by *β*-caryophyllene and *β*-caryophyllene oxide and the contribution to their chemosensitizing properties.

## 4. Materials and Methods 

### 4.1. Chemical and Reagents

All the chemicals, including *β*-caryophyllene (≥98.5% purity), *β*-caryophyllene oxide (95% purity), doxorubicin hydrochloride (98.0%–102.0% purity), rhodamine and ethanol (EtOH; ≥99.5% purity) were purchased from Sigma Aldrich Co (St. Louis, MO, USA). Dulbecco’s Modified Eagle’s Medium Ham’s F12 (DMEM-F12) was provided by Aurogene (Rome, Italy). The sources of antibodies and materials for molecular biology analysis were specified in the relative paragraphs. To perform the experiments, all solutions were prepared in the better solvent, sterilized and stored for a just conservation time at recommended temperature. The natural sesquiterpenes and doxorubicin were dissolved in EtOH 100% *v*/*v* and deionized water, respectively, and hence were diluted in the complete medium. In order to avoid any cytotoxicity, EtOH was used at a maximum concentration of 1% *v*/*v* in the medium.

### 4.2. Cell Culture 

Human hepatoma cell line HepG2 were obtained from American Type Culture Collection (ATCC). The cells were grown under standard conditions (37 °C and 5% CO_2_) in DMEM-F12 medium containing L-glutamine (1% *v*/*v*) and HEPES (15 mM) and were supplemented with 10% heat-inactivated FBS, 100 U/mL penicillin and 100 μg/mL streptomycin in 75 cm^2^ flasks. Cells were subcultured every 4 days, renewing growth medium twice a week, as recommended by the supplier.

### 4.3. Cytotoxicity Assay

The cultured cells were seeded into 96-well microplates (2 × 10^4^ cells/well), allowed to grow for 24 h, then treated with the test substances according to the time exposure required by scheduling protocols. Then, the cell viability was measured according to previous published methods [83]. Briefly, at the end of incubation, 10 μL of MTT solution (5 mg/mL) were added to each well and the plate was incubated at 37 °C for 80 min. The culture medium was then removed and 200 μL of DMSO was added to each well. The plate was stirred gently for 5 min, to dissolve the formazan product; then, the absorbance was detected at 595 nm by using a microplate reader (Epoch Microplate Spectrophotometer, BioTeK Instruments Inc., Winooski, VT, USA). The amount of formazan crystals formed was directly proportional to the viability of cells and the percentage growth inhibition by the compound was calculated. In order to obtain reproducible data, the assay was carried out at least three times and, in each experiment, each concentration was tested in triplicate, also including a vehicle control. The reduction of cell viability induced by the treatment was evaluated by comparing the number of viable cells of the vehicle control and that of the treatment. A treatment was considered cytotoxic when the cell viability was less than 70% with respect to the control [84].

### 4.4. Combination Assay

For evaluating the chemosensitizing properties, the anticancer drug and the chemosensitizing agents were administered to cells according to the co-treatment protocol. Two non-cytotoxic concentrations of the sesquiterpenes (about IC_10_ and IC_20_ concentrations, at which a 10% and 20% cytotoxicity was produced) were tested. Progressive concentrations of the anticancer drug were prepared in sterile tubes and gently mixed with the test substances or the vehicle. 

### 4.5. Scheduling of Single and Metronomic Treatment Protocols

The cells (2 × 10^4^ cells/well) were cultivated as described above, then exposed to the treatment under single and metronomic scheduling protocols. In the single long-term treatment, the cells were treated with the test substances for 24, 48 and 72 h, then the cytotoxicity was measured by MTT assay (Figure S1). Metronomic treatment was based on a short and/or repeated exposure of 2 h to the test substances, as follow: (1) Single treatment protocol, the cells were treated with the test substances for 2 h, then washed and incubated for 72 h (Figure S2); (2) Two-repeated treatment protocol, the cells were treated with the test substances for 2 h, then washed and incubated for functional recovery for further 2 h. After recovering the cells were subjected to a further 2 h treatment with the test substances, to a subsequent washing and finally to incubation for 72 h (Figure S2); (3) Three-repeated treatment protocol, the cells were treated as in the two-repeated treatment protocol but an additional treatment of 2 h with the test substances was performed before the 72 h incubation. Lastly, the cytotoxicity was measured by the MTT assay.

### 4.6. Analysis of Sesquiterpene-Drug Interactions

The type of interaction (synergistic, additive or antagonistic effect) was evaluated by reversal ratio value (RR), combination index (CI) and isobolographic analysis (IB), according to our previous published methods (Di Giacomo et al., 2017). Reversal ratio (RR), also known as cytotoxicity enhancement ratio, allows the quantification of the efficacy increase of a chemotherapeutic (A) in the presence of a chemosensitizer (B), by relating the IC_50_ of A alone (CA) with IC_50_ of the A and B combination (CA + B). The quantitative measurement of the interaction (namely CI) was calculated by the equation CI = (C_A,X_/IC_X,A_) + (C_B,x_/IC_X,B_), in which CA,X and CB,X are the concentrations of drugs A and B at the IC_50_ value of the combination, while ICX,A and ICX,B are the IC_50_ values of the drugs alone. The interaction was considered additive, synergistic and antagonistic when the CI value was equal to, less, or higher than 1, respectively.

Isobolographic analysis shows the extent of the interaction between the potential chemosensitizer (A) and the chemotherapeutic (B). The IC_50_ concentrations of drugs A and B were plotted on the x and y axes in a two-coordinate plot, corresponding to (CA, 0) and (0, CB), respectively. The line connecting these points represented an additive interaction. The concentrations of the drugs used in combination (CA and CB) are placed in the same plot. In order to connect the IC_50_ value of each drug alone and that of the combination, a nonlinear regression analysis (equation y = (top – bottom) × exp(–kx) + bottom) has been carried out by GraphPad Prism™ 6.00 software. The effect was synergistic when CA and CB are located below the line, while antagonistic when the values are above the line [27].

### 4.7. Doxorubicin Accumulation

Cells were cultured on 6-well plates (1 × 10^6^ cells/well) and treated with the tested sesquiterpenes (5, 50 and 100 μM) for 2 h before adding doxorubicin. Thereafter, the cells were harvested and washed, and the intracellular doxorubicin accumulation was measured by flow cytometry at the FL1 emission spectrum (485 nm excitation wavelength; 528 nm emission wavelength) by a BD Accuri™ C6 flow cytometer (BD Biosciences, Italy). 

### 4.8. Rhodamine 123 Efflux Assay

P-gp efflux was evaluated by the rhodamine 123 assay, according to previous published methods [16], using verapamil as a standard P-gp inhibitor. Briefly, confluence cells (1 × 10^6^ cells/well) were subjected to a pre-treatment with the test substances or the positive control verapamil (5, 50 and 100 μM) for 2 h, then rhodamine 123 was added. After harvesting and washing, the rhodamine 123 fluorescence was measured at the FL1 emission spectrum (485 nm excitation wavelength; 528 nm emission wavelength) by a BD Accuri™ C6 flow cytometer. 

### 4.9. Western Blotting Analysis

The analysis was made according to a previously published method [85]. Cells were cultured on 6-well plates, treated with caryophyllane sesquiterpenes (50 µM) and a low dose of doxorubicin (20 µM) for 2 h, alone and in combination, then harvested by centrifugation and washed in PBS. Cell proteins were separated by a lysis buffer, containing SDS (2% *w*/*w*), Tris-hydrocloride (20 mM; pH 7.4), urea (2 M), glycerol (10% *w*/*w*), sodium orthovanadate (2 mM), DTT (10 mM), and a protease inhibitors cocktail (1:100 dilution). Proteins were resolved by SDS-PAGE 10% TGX FastCast™ Acrylamide gel (BioRad, Segrate, Italy) and transferred on PVDF membranes (BioRad, Segrate, Italy) using Trans-Blot^®^ Turbo™ Transfer System (BioRad, Segrate, Italy). The membranes were blocked with 0.2% *w*/*v* I-block (Thermo Fisher Scientific, Rodano, Italy) in Tris-buffered saline containing 0.05% Tween-20 (TBS-T) and incubated with the P-gp primary antibody (anti-MDR1/ABCB1 rabbit antibody; mAB #13342 from Cell Signaling Technology, Euroclone, Pero, Italy) for 1 h. Subsequently, membranes were washed three times in TBS-T, and then incubated for an additional hour with appropriate horseradish peroxidase or alkaline-phosphatase-conjugated secondary antibody (Jackson ImmunoResearch, Pero, Italy). The peroxidase signal was detected with ECL Fast Femto reagent (Immunological Science, Roma, Italy), acquired by Molecular Imager^®^ ChemiDoc™ MP System (Bio-Rad, Segrate, Italy), and the intensity of protein bands was quantified using ImageJ Software. The alkaline phosphatase signal was detected with BCIP/NBT reagents (Carl Roth, Milano, Italy, CAS No. 298-83-9 and 6578-06-9). *β*-actin (total extracts) was used as normalization protein. Each experiment was replicated at least three times.

### 4.10. Immunofluorescence

The analysis was performed according to previously published methods [86]. Briefly, the cells were seeded on a coverslip in a six-well plate and allowed to adhere overnight, then subjected to different treatments with the caryophyllane sesquiterpenes (50 µM) and a low dose of doxorubicin (20 µM) for 2 h, alone and in combination, and fixed onto slides by incubation in pure methanol for 2 min. Then, slides were washed by PBS (1 X), further incubated in 4% bovine serum albumin (BSA) and PBS + Tween 20 (PBS-T), and then added with P-gp primary (anti-MDR1/ABCB1 rabbit antibody; mAB #13342 from Cell Signaling Technology) antibody for 1 h at room temperature. After the reaction with the secondary antibody (donkey anti-rabbit antibody Alexa Fluor 488; A-21206 from Invitrogen Thermo Fisher Scientific) for 45 min in a dark room and RT, the cells were rinsed with PBS-T and the coverslips were put onto slides with a drop of DAPI. Slides were examined to analyze the cellular expression of P-gp and localization was carried out using Leica Microsystems DM 4500 B Light and Fluorescence Microscopy (Weltzlar, Germany) equipped with a JenoptikProg-Res-C10 Plus Videocam (Jena, Germany). A semiquantitative analysis of the P-glycoprotein (P-gp) expression in HepG2 cells was carried out (four fields for each treatment) according to a previous published grading system [31], as follow: negative, <5%; +/−, 6%–10%; +, 11%–30%; ++, 31%–60%; +++, >61%.

### 4.11. In Silico Prediction of the P-Glycoprotein Active Site for Caryophyllane Sesquiterpenes 

#### 4.11.1. Docking and Active Site Prediction

*β*-caryophyllene and *β*-caryophyllene oxide and the steroisomer *α*-caryophyllene (or *α*-humulene), were energy minimized in Chemoffice 16.0 (PerkinElmer Informatics, Inc., Shelton, CT, USA) by PM3. The recently published human homology model of P-glycoprotein [26] was used for blind docking and active site prediction. Docking was performed by AutoDock vina and the whole structure of the protein was considered for evaluation of potential binding sites. The center of the grid was set to X = 90.07, Y = 145.72, Z = 107.05 and the grid size was 116, 100 and 72 in the X, Y and Z dimensions. 

#### 4.11.2. Molecular Dynamic Simulation and Binding Free Energy Calculation

GROMACS version 2019 installed on an Ubuntu 18.04 Linux workstation with intel Core i7 cpu (8 x 3.20 GHz) was used for dynamic simulation of compounds in the P-gp binding site in explicit water and a gromos96 54A7 forcefield was used for topology generation. Topology of ligands was generated by the ATB server and atomic charges were modified using PM3 or Muliken atomic partial charges calculated in Gaussian 09. The topology and coordinate files for the protein were generated using pdb2gmx program of GROMACS package taking parameters from the gromos96 54A7 forcefield. The coordinate and topology files of the protein and the ligands were then merged to obtain the final starting structure and topology file for each complex.

The complex was centered in a dodecahedron periodic box and solvated by the addition of water molecules (simple point charge model). The total charge of the system was then neutralized by the addition of sodium and chloride ions as required. Sequentially, energy minimization was performed by the steepest descent algorithm. The system was then gradually heated to 300 K and was equilibrated at 100 ps using the NVT (constant volume and temperature) ensemble with position restraint for the heavy atoms, followed by 100 ps equilibration in the NPT (constant pressure and temperature) ensemble at 1 atm. Both temperature and pressure were regulated using the Berendsen algorithm. Finally, the full system was subjected to 1 ns MD simulation with a 2 fs time-step interval (Figure S6). The temperature and pressure were maintained at 300 K and 1 atm using the v-rescale temperature and Parrinello-Rahman pressure coupling method. The short-range non-bonded interactions were computed for the atom pairs within the cut-off of 1.2 nm, while the long-range electrostatic interactions were calculated using Particle-Mesh-Ewald summation method with fourth-order cubic interpolation and 1.2 Å grid spacing. All h-bonds were constrained using the parallel LINCS method. 

Lastly, MM-PBSA method for calculating free energy of binding was done by g-mmpbsa script introduced by Kumari et al. [87]. g-mmpbsa tool is an open source tool written in the C programming language and does not depend on any external software. This tool contains all the required subroutines from the GROMACS and the APBS (adaptive Poisson-Boltzmann solver) packages to calculate the enthalpic components of the MM-PBSA (molecular mechanics Poisson-Boltzmann surface area) interaction [88].

### 4.12. Statistical Analysis

Data were analyzed by GraphPad Prism TM (Version 6.00) software (GraphPad Software, San Diego, CA, USA) and expressed as the mean ± SE (standard error) of at least two experiments in which each treatment was tested in triplicate. The one-way analysis of variance (one-way ANOVA), followed by Dunnett’s multiple comparison post-test, were used to verify the level of significance regarding the comparison of the response with respect to control. The concentration–response curves were constructed using the “Hill equation”: E = E_max_/ [1 + (10^LogEC50^/A)^HillSlope^], where E is the effect at a given concentration of the substance, E_max_ is the maximum activity, IC_50_ is the concentration that produces a 50% of the inhibitory response, A is the substance concentration, HillSlope is the curve slope. The *p* values < 0.05 were considered as significant.

## 5. Conclusions

The present study demonstrated that caryophyllane sesquiterpenes are able to potentiate the cytotoxicity of low-dose doxorubicin both under standard and metronomic schedules. This is an interesting goal as it allows the achievement of successful chemotherapy whilst limiting the side effects which often lead to early treatment suspensions due to the low tolerability by patients. Moreover, the interference of the P-gp transporter by caryophyllane sesquiterpenes has been highlighted to be involved in their chemosensitizing properties, although it appears to be a result of multiple mechanisms, including: a direct protein inhibition at the binding site, likely due to a peculiar chemical feature of the caryophyllane scaffold, i.e., an indirect inactivation probably to be ascribed to an alteration of the membrane permeability; and a regulation of the protein expression in which a contribution of STAT3 cascade could be hypothesized. Therefore, caryophyllane sesquiterpenes appear to possess pleiotropic effects which deserve to be better elucidated in order to evaluate an interest for possible pharmacological applications.

In conclusion, present findings strengthen our hypothesis about the potential chemosensitizing power of caryophyllane sesquiterpenes and suggest that combining a chemosensitizer and metronomic schedule can represent a suitable strategy to overcome the drawbacks of doxorubicin chemotherapy while exploiting its powerful activity. 

## Figures and Tables

**Table 1:** IC_50_ values of doxorubicin and caryophyllane sesquiterpenes under both long-term and metronomic schedules. In the last protocol, the cells were subjected to a short and/or repeated exposure of 2 h to the test substances followed by a recovery time of 72 h. Data represent the mean ± SE (standard error) of at least two experiments in which each treatment was tested in triplicate (*n* = 6).

Time Exposure	IC_50_ [µM] (CL)		
	Doxorubicin	*β*-Caryophyllene	*β*-Caryophyllene Oxide
24 h	14.4 (12.8–16.2)	197.0 (127.0–314.5)	195.0 (172.5–219.5)
48 h	3.6 (2.6–5.2) ^§^**	121.0 (94.0–159.5) ^§^	162.0 (146.0–180.0)
72 h	1.6 (1.4–1.8) ^§^**	113.0 (88.5–144.0) ^§^	152.5 (136.5–170.0)
2 h	13.6 (2.6–20.2)	379.5 (171.5–460.5)	354.5 (190.5–441.0)
2 h double	11.6 (6.4–20.4) ^§^*	265.5 (110.5–327.0)	281.0 (157.5–360.5)
2 h triple	5.8 (2.8–11.6) ^§^**	251.0 (71.5–372.0)	256.5 (123.5–334.0)

CL, confidential limits. ^§^ *p* < 0.01 (ANOVA + multiple Dunnett’s comparison post-test), significantly lower than the IC_50_ value obtained after 24 h exposure. * *p* < 0.05 and ** *p* < 0.01 (ANOVA + multiple Dunnett’s comparison post-test), significantly lower than the IC_50_ value after the single short treatment of 2 h.

**Table 2:** IC_50_ values of doxorubicin and its combination with *β*-caryophyllene or *β*-caryophyllene oxide under the scheduled exposure protocols. Data represent the mean ± SE (standard error) of at least two experiments in which each treatment was tested in triplicate (*n* = 6).

Time Exposure	IC_50_ [µM] (Confidential Limits) *RR* ^a^				
	Doxorubicin	Doxorubicin + *β*-Caryophyllene [50 µM]	Doxorubicin + *β*-Caryophyllene [100 µM]	Doxorubicin + *β*-Caryophyllene Oxide [50 µM]	Doxorubicin + *β*-Caryophyllene Oxide [100 µM]
24 h	14.4 (12.8–16.2)	8.6 (7.6–13.8) ** 1.7	1 (0.4–1.8) *** 14.4	5.0 (4.2–5.8) *** 2.9	3.4 (2.2–4.6) *** 4.2
48 h	3.6 (2.6–5.2)	3.2 (2.0–5.2) 1.4	2.8 (1.2–5.2) 1.7	3.2 (1.6–5.2) 1.6	2.8 (1.2–4.8) 1.8
72 h	1.6 (1.4–1.8)	1.0 (0.8–1.4) 1.6	1.2 (0.6–3.4) 1.3	1.0 (0.8–1.6) 1.6	1.2 (0.6–2.6) 1.3
2 h	13.6 (2.6–20.2)	8.2 (3.2–21.4) ** 1.7	3.6 (1.8–7.2) *** 3.8	6.6 (2.4–18.0) ** 2.1	8.6 (5.0–14.0) ** 1.6
2 h double	11.6 (6.4–20.4)	3.2 (1.4–7.0) ** 2.6	4.6 (2.2–9.6) ** 1.8	2.2 (0.2–8.4) *** 3.7	5.6 (2.6–12.2) ** 1.5
2 h triple	5.8 (2.8–11.6)	3.2 (1.8–5.4) * 1.8	3.4 (2.0–6.6) * 1.7	4.6 (3.0–6.2) 1.3	5.4 (3.6–9.0) 1.1

^a^ Reversal ratio (RR) represents the ratio between the IC_50_ values of doxorubicin and its combination with *β*-caryophyllene and *β*-caryophyllene oxide. * *p* < 0.05, ** *p* < 0.01 and *** *p* < 0.001 (ANOVA + multiple Dunnett’s comparison post-test), significantly lower than doxorubicin in the same time schedule.

**Table 3:** Mean binding energy and its decomposed constituent’s energies (kJ/moL) for verapamil and the caryophyllane sesquiterpenes in the predicted binding site of P-gp calculated by MM-PBSA method.

Compound	Van Der Waals Energy	Electrostatic Energy	Polar Solvation Energy	SASA Energy	Mean Binding Energy
*α*-Caryophyllene	−133.59	23.59	49.38	−12.40	−73.01
*β*-Caryophyllene	−108.27	−0.22	32.02	−12.00	−88.46
*β*-Caryophyllene oxide	−106.92	−2.60	39.94	−12.98	−82.57
Verapamil	−212.66	−11.34	104.62	−23.58	−142.97

## Supplementary Materials

Supplementary materials can be found at https://www.mdpi.com/1422-0067/21/2/633/s1, Figure S1: Schedule of the long-term exposures of 24, 48 and 72 h. Figure S2: Schedule of the metronomic treatment. Figure S3: Effect of the natural sesquiterpenes *β*-caryophyllene and *β*-caryophyllene oxide in combination with the anticancer drug cisplatin in HepG2 cells after 24 and 72 h exposure. Figure S4: Contribution energy of each amino acid to the binding energy of verapamil in kJ/mol. Figure S5: 2D-map of verapamil in the binding site of P-gp representing interactive amino acids and types of interactions. Figure S6: RMSD (Root-mean-square deviation) curve of the protein during 1 ns dynamic simulation. Table S1: Contribution energy values per the most important residues in kJ/mol for the sesquiterpenes *β*-caryophyllene and *β*-caryophyllene oxide and for the standard control verapamil.
