# Supplementary material for: Potentiation of Low-Dose Doxorubicin Cytotoxicity by Affecting P-Glycoprotein through Caryophyllane Sesquiterpenes in HepG2 Cells: An In Vitro and In Silico Study
Source: Int J Mol Sci. 2020 Jan 17;21(2):633. doi: 10.3390/ijms21020633 (PMC7014471; doi:10.3390/ijms21020633)
Supplement: Supplementary file 1 [file ijms-21-00633-s001.pdf]

Supplementary materials

Potentiation of low-dose doxorubicin cytotoxicity by affecting P-glycoprotein through caryophyllene sesquiterpenes in HepG2 cells: an *in vitro* and *in silico* study

Antonella Di Sotto [1,*], Hamid Irannejad [2], Margherita Eufemi [3], Romina Mancinelli [4], Lorena Abete [1], Caterina Loredana Mammola [4], Fabio Altieri [3,*], Gabriela Mazzanti [1], Silvia Di Giacomo [1]

[1] Department of Physiology and Pharmacology "V. Erspamer", Sapienza University of Rome, P.le Aldo Moro 5, 00185 Rome, Italy. antonella.disotto@uniroma1.it; lorena.abete@uniroma1.it; gabriela.mazzanti@uniroma1.it; silvia.digiacomo@uniroma1.it

[2] Department of Medicinal Chemistry, Faculty of Pharmacy, Mazandaran University of Medical Sciences, Sari, Iran. irannejadhamid@gmail.com

[3] Department of Biochemical Science "A. Rossi Fanelli", Sapienza University of Rome, P.le Aldo Moro 5, 00185 Rome, Italy. fabio.altieri@uniroma1.it; margherita.eufemi@uniroma1.it

[4] Department of Anatomical, Histological, Forensic and Orthopedic Sciences, Sapienza University of Rome, P.le Aldo Moro 5, 00185 Rome, Italy. romina.mancinelli@uniroma1.it; caterinaloredana.mammola@uniroma1.it

* Correspondence: antonella.disotto@uniroma1.it (A.D.S.) and fabio.altieri@uniroma1.it (F.A.)

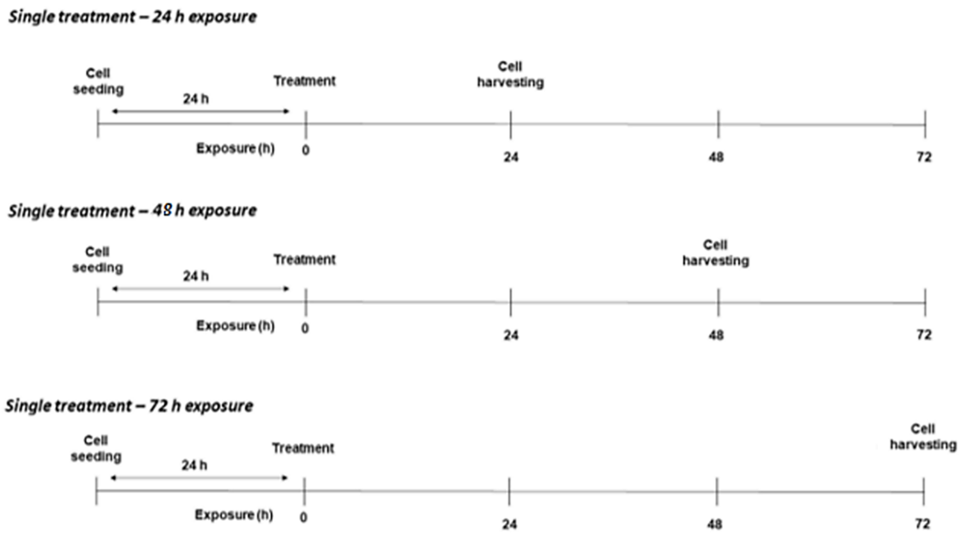

Figure S1. Schedule of the long-term exposures of 24, 48 and 72 h.

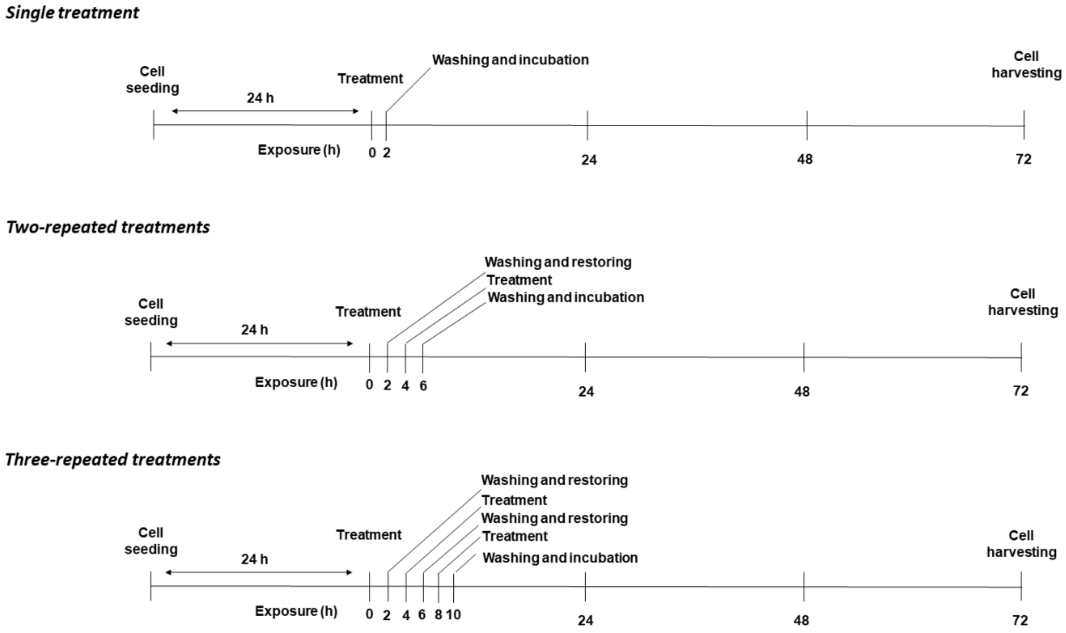

Figure S2. Schedule of the metronomic treatment. The cells were subjected to a short and/or repeated exposure of 2 h followed by a recovery time of 72 h.

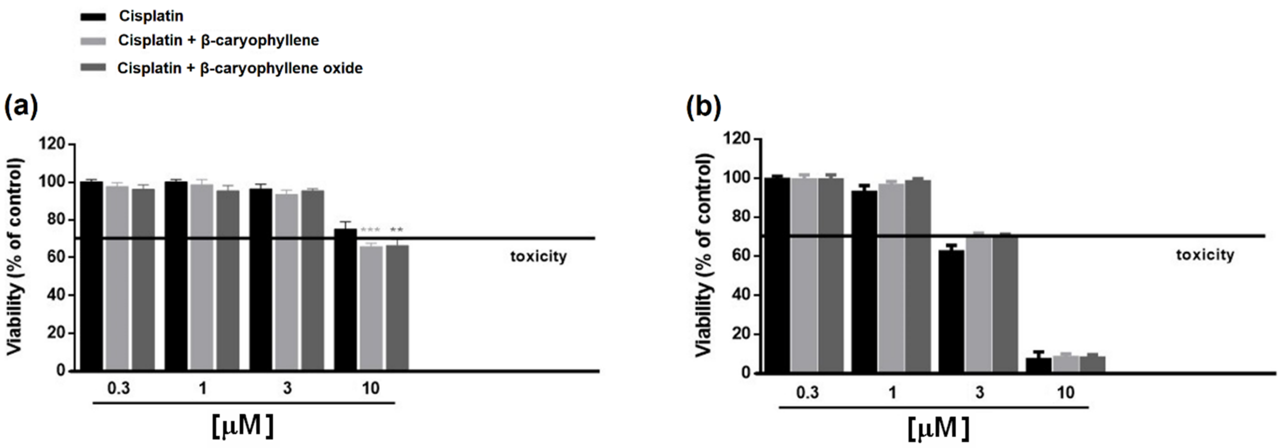

Figure S3. Effect of the natural sesquiterpenes β-caryophyllene and β-caryophyllene oxide (50 μM) in combination with the anticancer drug cisplatin (0.3-10 μM corresponding to 0.1-3 μg/mL) in HepG2 cells after 24 (a) and 72 h (b) exposure. After 24 h exposure, cisplatin induced early signs of cytotoxicity (about 20% inhibition of cell viability) at the highest concentration tested, whereas biologically significant cytotoxic effects were registered after a long-term exposure of 72 h, achieving a maximum 90% inhibition of cell viability at the highest tested concentration. The combination of cisplatin with the caryophyllene sesquiterpenes did not increase the anticancer drug cytotoxicity in all the experimental conditions.

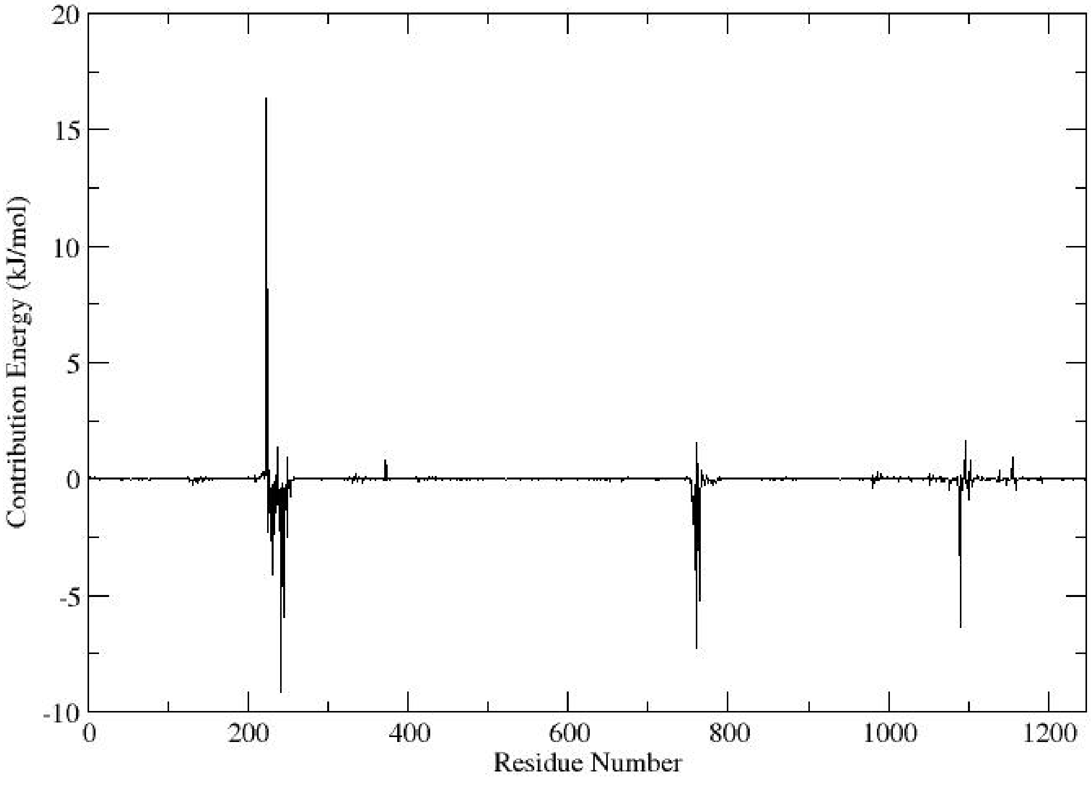

Figure S4. Contribution energy of each amino acid to the binding energy of verapamil in kJ/moL.

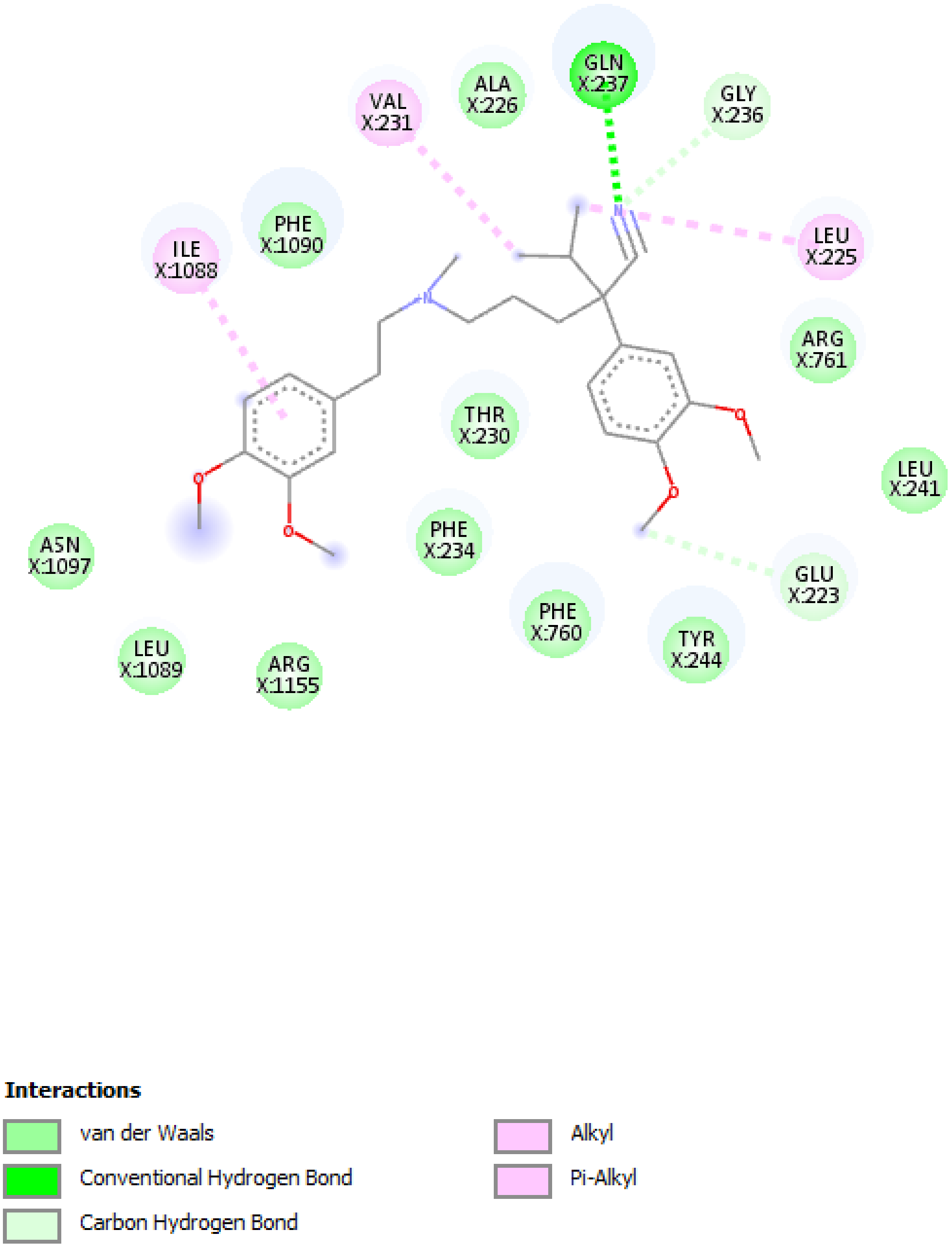

Figure S5. 2D-map of verapamil in the binding site of P-gp representing interactive amino acids and types of interactions.

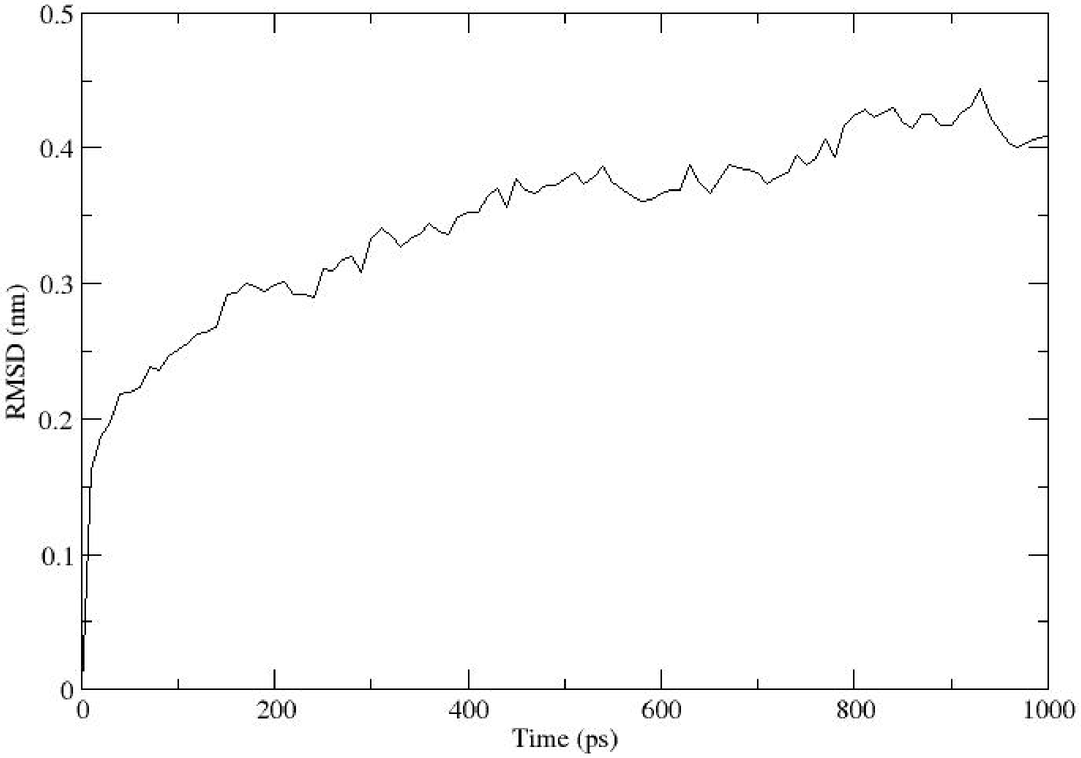

Figure S6. RMSD (Root-mean-square deviation) curve of the protein during 1 ns dynamic simulation.

Table S1. Contribution energy values per the most important residues in kJ/mol for the sesquiterpenes β-caryophyllene and β-caryophyllene oxide and for the standard control verapamil (only residues with high favoured/unfavoured energy values are presented).

Residue	β-Caryophyllene	β-Caryophyllene oxide	Verapamil
Glu223	+2.2	+2.2	+16.3
Leu225	-5.0	-3.9	-2.2
Ala226	+1.3	+1.3	----
Ile228	-3.2	-1.8	-2.6
Val231	-3.5	-3.3	-4.1
Phe234	-----	-----	-1.4
Gly236	-----	-----	-1.1
Gln237	-----	+1.4	+1.3
Lys238	-----	-----	-2.2
Leu241	-----	-1.9	-9.1
Arg243	----	-----	-1.5
Tyr244	-----	-----	-5.9
Leu248	-----	-----	-2.5
Lys372	+2.6	-----	----
Tyr757	----	-----	-1.2
Phe760	-2.6	-3.3	-7.2
Arg761	----	+1.0	+1.5
Met763	-----	-----	-1.0
Leu764	-3.4	-2.3	-5.2
Trp770	-1.5	-----	-----
Phe771	-1.5	-1.6	-----
Thr777	-----	+1.2	-----
Thr778	-----	-1.7	-----
Leu781	-2.2	-2.5	-----
Phe1090	-----	-----	-6.3
Glu1096	-----	-----	+1.6
